# Off-Stoichiometry Thiol–Ene Polymers: Inclusion of Anchor Groups Using Allylsilanes

**DOI:** 10.3390/polym15061329

**Published:** 2023-03-07

**Authors:** Kirill Puchnin, Dmitriy Ryazantsev, Egor Latipov, Vitaliy Grudtsov, Alexander Kuznetsov

**Affiliations:** 1 Scientific-Manufacturing Complex Technological Centre, Zelenograd 124498, Russia; 2 Institute of Nanotechnology of Microelectronics of the Russian Academy of Sciences, Moscow 119991, Russia

**Keywords:** OSTE, silane, thiol–ene, photocuring, bonding, microfluidic, integration

## Abstract

The use of polymers in silicon chips is of great importance for the development of microelectronic and biomedical industries. In this study, new silane-containing polymers, called OSTE-AS polymers, were developed based on off-stoichiometry thiol–ene polymers. These polymers can bond to silicon wafers without pretreatment of the surface by an adhesive. Silane groups were included in the polymer using allylsilanes, with the thiol monomer as the target of modification. The polymer composition was optimized to provide the maximum hardness, the maximum tensile strength, and good bonding with the silicon wafers. The Young’s modulus, wettability, dielectric constant, optical transparency, TGA and DSC curves, and the chemical resistance of the optimized OSTE-AS polymer were studied. Thin OSTE-AS polymer layers were obtained on silicon wafers via centrifugation. The possibility of creating microfluidic systems based on OSTE-AS polymers and silicon wafers was demonstrated.

## 1. Introduction

The use of polymer materials for the manufacture of microfluidic devices and their integration with complementary metal–oxide–semiconductor (CMOS) biosensors provides simplicity and cost effectiveness for both lab-on-a-chip devices and microanalysis systems (µTAS) [1,2,3]. When integrating chips with microfluidic systems, many problems arise related to the separation of the liquid and electrical interfaces, sealing, and the functionalization of the channel surface [4]. Poly(dimethylsiloxane) (PDMS) has become one of the main materials used for the low-volume production of microfluidic devices due to its low cost and ease of handling [5,6]. It is a soft polymer, on the basis of which various components of microfluidic systems have been produced, such as mixers, pneumatically actuated switches and valves, magnetic filters, functional membranes, and optical components [7]. However, when switching from prototyping to commercial samples, PDMS is often not suitable due to its low mechanical strength and slow thermal curing.

Off-stoichiometry thiol–ene (OSTE) polymers have been proposed to replace PDMS. The basis for the production of these polymers is a simple “click” reaction, i.e., the interaction of the functional groups of two monomers: one containing several allyl fragments and the other containing several thiol fragments. Polymer curing occurs under UV irradiation due to the presence of a UV initiator of a radical reaction in the prepolymer mixture. Varying the ratio of monomers allows for the mechanical properties of OSTE polymers to be adjusted, such as the Young’s modulus and the glass transition temperature, as well as for polymers with an excess of thiol or allyl groups to be obtained [8]. Polymer pieces with an excess of thiol and allyl groups can be subjected to direct UV bonding, which can be used to create multilayer microfluidic systems [9].

Such extensive prototyping capabilities have made it possible to use OSTE polymers in various fields of science and technology, e.g., in microelectronics to create flexible electrodes [10] and the gate dielectric of organic thin-film transistors [11], in analytical chemistry for separation and concentration [12,13,14], in photonics for encapsulation [15,16,17], etc. Moreover, silicon fragments can be included in the composition of OSTE polymers. The use of silicon-based vinyl monomers has made it possible to obtain materials with high refractive indices and Abbe numbers [18]. The inclusion of silicon in the composition of thiol monomers allows one to achieve an improvement in the storage stability of formulations and an increase in the glass transition temperature compared to that of samples that are prepared with pentaerythritol tetra-3-mercaptopropionate (PETMP) [19]. Adding silane modifiers to corrosion-resistant coatings based on thiol–ene improves their affinity to wet (spread on) surfaces [20].

It is possible to bind OSTE polymers to silicon surfaces. Until recently, two methods of binding polymers to silicon wafers had been described. In the first method, the surface of silicon wafers is modified in advance with silanes containing terminal vinyl or isocyanate groups, after which they interact with the excess thiol groups of the polymer [21]. In the second method, a modified OSTE polymer, including a reagent containing epoxy groups (for example, bisphenol A diglycidyl ether), is used. After heating this polymer, the epoxy ring opens, which leads to the bonding of the polymer with a silicon surface and a significant conversion of the mechanical properties of the polymer [22].

Our scientific group proposed a third method that makes it possible to bind OSTE polymers with silicon wafers. Silane groups are included in the polymer using (3-mercaptopropyl)trimethoxysilane, which reacts with the allyl monomer during polymerization. Then, the resulting polymer, named OSTE-MS, is bonded with silicon wafers after heating [23]. It is worth noting that this method has a number of advantages. Unlike the first method of bonding, the surface of the silicon wafer does not need to be additionally modified, and unlike the second method, the mechanical properties of the polymer change only slightly after the binding stage.

**Figure 1:**
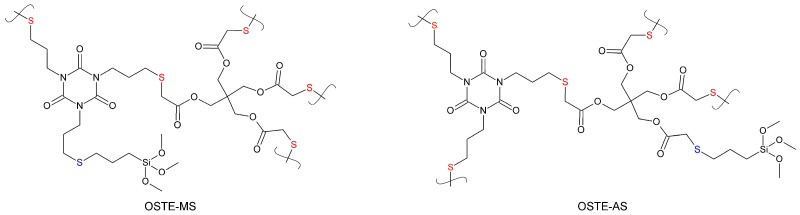
The silane-containing fragments of OSTE-MS and OSTE-AS polymers. The sulfur bridges between monomer fragments are indicated in red. The sulfur bridges between monomer fragments and silanes are indicated in blue.

In this work, we continue our research on the inclusion of silane groups in the polymer framework. OSTE polymers are composed of two monomers: one containing thiol groups and the other containing allyl groups. The inclusion of silane groups is possible using both thiol and allyl monomers. It is worth noting that the monomers have a different number of functional groups; therefore, when binding silanes in the case of allyl monomers, only two bonds remain for the inclusion of this fragment in the polymer framework (Figure 1, OSTE-MS), which leads to the appearance of linear fragments in the polymer. In the case of the modification of thiol monomers by silanes, three thiol groups remain free, which makes it possible to form a net with these monomers (Figure 1, OSTE-AS). Such a difference in structure could lead to a difference in the mechanical properties of polymers, which requires study. In our previous work, we studied OSTE polymers in which silane was included in an allyl monomer. In this work, we studied OSTE polymers in which silane was included in a thiol monomer. In addition, the inclusion of silanes in the thiol monomer simplifies the production of silane-containing OSTE polymers, which have an excessive number of allyl groups, and the inclusion of silanes into the allyl monomer simplifies the preparation of thiol-excessive OSTE polymers. Obtaining thiol- or allyl-excessive polymers can be useful for their further selective modification by various reagents, including when they are found together in the same product, and for the crosslinking of thiol- and allyl-excessive polymers with each other.

## 2. Materials and Methods

### 2.1. Materials

All chemicals obtained from commercial sources were used without further purification. Pentaerythritol tetrakis(mercaptoacetate) (PETMA, 90%, CAS: 10193-99-4), triallyl isocyanurate (TATATO, 98%, CAS: 1025-15-6), and 2,4,6-trimethylbenzoylphenylphosphinic acid ethyl ester (TPO-L, 95%, CAS: 84434-11-7) were purchased from ABCR (Karlsruhe, Germany). Allyltrimethoxysilane (AS, 95%, CAS: 2551-83-9) was purchased from Sigma-Aldrich (St. Louis, MO, USA). The (100) P-type KDB10 silicon-on-insulator wafers were purchased from Telecom-STV Company Limited (Zelenograd, Russia). Solvents were purified and dried according to standard procedures.

### 2.2. OSTE-AS Preparation

The general preparation method of OSTE-AS polymers is as follows: 6 mL (27.1 mmol) TATATO, 6 mL (17.4 mmol) PETMA, 1 mL (5.7 mmol) AS, and 0.06 mL (0.2 mmol) TPO-L were mixed at room temperature using a rotator at 30 rpm for 15 min. A liquid prepolymer was poured into a mold. The sample was irradiated with UV LED (365 nm, 10 W) for 1 min per 1 mm thickness of the OSTE-AS polymer layer. Then, the sample was bonded to the silicon surface (with a natural oxide), applying 110 °C heat for 15 min. To create better contact between the polymer and the silicon wafer, a pressure of about 0.5 MPa was applied. No pressure was applied to samples that did not require bonding, but heat treatment was applied. When optimizing the polymer composition, the ratio of reagents was varied.

### 2.3. Characterization

Most of the methods used to study the physicochemical properties of the polymers were adopted from our previous work [23] without changes. This allows for a direct comparison of the results.

Fourier transform infrared (FT-IR) spectra were recorded on a Nicolet iS50 FT-IR with a diamond crystal attenuated total reflection (ATR) accessory (Thermo Scientific, Waltham, MA, USA). A total of 64 scans per measurement were carried out.

NMR spectra were acquired at 25 °C on an AVANCE 400 (Bruker, Bremen, Germany) spectrometer. Extraction was carried out from an OSTE-AS polymer sample weighing 30 mg with 0.7 mL of CDCl_3_ for 1 h. A total of 32 scans per measurement were carried out.

The hardness of the polymers was measured on a Digital Shore Durometer Type D (Dongying, Shandong, China) with the use of a mechanical stand. Polymer cubes with dimensions of 9.5 × 9.5 × 9.5 mm were used for the measurements. The result was recorded after 15 s of contact between the durometer and the polymer. The final result was taken as the average of three measurements.

The tensile strain of the OSTE-AS polymers under tension was measured on an AGS-10 (Shimadzu, Kyoto, Japan) universal testing machine at room temperature. The samples were cast in a 3D-printed PLA mold. The cross section of the sample was 2 × 4 mm. The distance between the holders was 35 mm. The test speed was 2 mm/min. The final result was taken as the average of three measurements.

The shift force of the polymeric columns was measured using an AMF-200 digital force gauge (ALIYIQI Instrument, Beijing, China). OSTE-AS polymer columns 4 mm in height and 1.65 × 1.65 mm wide were made on a silicon wafer pretreated with UV/ozone (BioForce Nanosciences, Ames, IA, USA). A force parallel to the plane of the wafer was applied to the columns. The shift force was equal to the maximum force at which the column came off the wafer. The final result was taken as the average of three measurements.

Contact angle measurements were conducted at room temperature using an OCA 15EC (DataPhysics Instrument, Filderstadt, Germany). A prepolymer or deionized water (electrical resistance = 18.2 MΩ cm) was dropped onto each surface, and the contact angles were assessed using SCA 20 software (Version 4.3.19).

The deposition of the OSTE-AS prepolymers on silicon wafers via centrifugation was carried out using an Apogee spin-coater module of an X-Pro II workstation (CEE, Saint James, MO, USA) at various rotation speeds. After UV irradiation and polymer hardening, the film thickness was measured using a SENDURO ellipsometer (SENTECH Instruments GmbH, Berlin, Germany).

The viscosity of the prepolymer mixture was measured on a Discovery HR-1 hybrid rheometer (TA Instruments, New Castle, DE, USA). For measurements, the prepolymer mixture was used without the addition of TPO-L UV initiator to prevent the premature polymerization of the sample. A 20 mm parallel plate was used for geometry. A gap of 1 mm was used.

UV-visible absorption spectra were recorded on an Infinite M200pro spectrophotometer (Tecan, Männedorf, Switzerland) using trUView Cuvettes (Bio-Rad Laboratories, Hercules, CA, USA). The prepolymer mixture was subjected to polymerization directly in the cuvette.

The dielectric permittivity was determined based on capacitance measurements with a variation in the upper contact area of the capacitor (the area of a drop of liquid metal) [24]. The capacitance was measured and calculated using a built-in module for quasistatic C-V measurements in B1500A (Agilent Technologies, Santa Clara, CA, USA) in the voltage range of −5 V to 5 V. Contact with the upper plate was made through a probe lowered into liquid metal (gallium), and contact with the lower plate was made through a substrate grounded on the probe station table.

Simultaneous thermogravimetry (STA) was carried out using an STA449 F1 Jupiter thermo-analyzer (Netzsch, Selb, Germany) with a Pt/Rh thermocouple. The calibration of sensitivity, temperature, and τ-rad was used for the entire heat range of 40–800 K. Measurements were carried out at a heat rate of 10 K/min with an air/argon (50, 20 mL/min) current. The temperatures of the phase transactions were determined using Proteus software (Version 8.0.2). Alumina crucibles were used.

To measure the chemical resistance, polymer cubes 9.5 × 9.5 × 9.5 mm in size were used. The chemical resistance was evaluated using three parameters: hardness, geometric dimensions, and weight. The parameters were measured after one hour, one day, and one week of conditioning. According to the totality of changes in these parameters, the solvents were divided into four nominal groups: good, normal, satisfactory, and unsatisfactory chemical resistance.

### 2.4. Formation of Microfluidic System

A Vaseline–wax sacrificial layer was applied to the silicon wafers using an EFD Ultimus V fluid dispenser (Nordson, Westlake, OH, USA). Next, the outer form (printed using a 3D printer) and connectors were joined. After the formation of the structure, it was filled with the prepolymer, irradiated with ultraviolet light, put under pressure, and heated for 15 min at 110 °C. The sacrificial layer was removed via double sonication with white spirit and double washed using hexane.

## 3. Results and Discussion

### 3.1. Preparation of OSTE-AS Polymers

**Figure 2:**
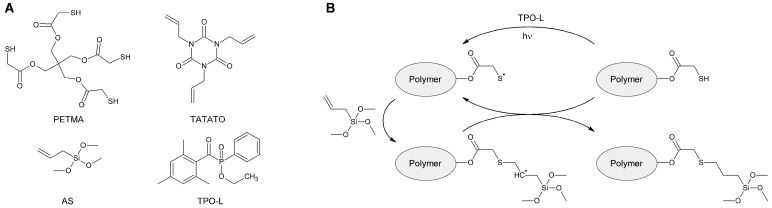
(**A**) Reagents used for the preparation of OSTE-AS polymers. (**B**) Scheme of inclusion of silane fragments into the polymer.

Pentaerythritol tetrakis(mercaptoacetate) (PETMA) and triallyl isocyanurate (TATATO) used in our previous work were selected as monomers for the preparation of modified OSTE polymers (Figure 2A). They completely dissolve in each other, and they dissolve various silanes well, which has a beneficial effect on the preparation of a homogeneous polymeric mass. In this work, it was decided to include silane groups in the polymer by using silanes containing terminal vinyl fragments. Allyltrimethoxysilane was chosen for this purpose. It is readily available, and the silane fragment has a lower reactivity than its trichlorosilane analogue. For example, it is less susceptible to hydrolysis. This simplifies working with it and allows one to increase the intervals between operations. In addition, methanol, which is formed during the hydrolysis of allyltrimethoxysilane, is friendly to many materials, unlike hydrochloric acid released during the hydrolysis of trichlorosilanes. This makes it possible to use the polymer developed in this work in various fields, for example, in microelectronics. TPO-L was used as a photoinitiator of radical polymerization. When the reaction mixture was irradiated with ultraviolet light, TPO-L disintegrated to form radicals that interacted with the thiol groups of PETMA or the formed polymer (Figure 2B). This led to the formation of thiyl radicals, which then interacted either with TATATO, which led to the growth of the polymer, or with allyltrimethoxysilane, which led to the inclusion of silane groups in the polymer. The polymer modified with allylsilanes was named the OSTE-AS polymer.

**Figure 3:**
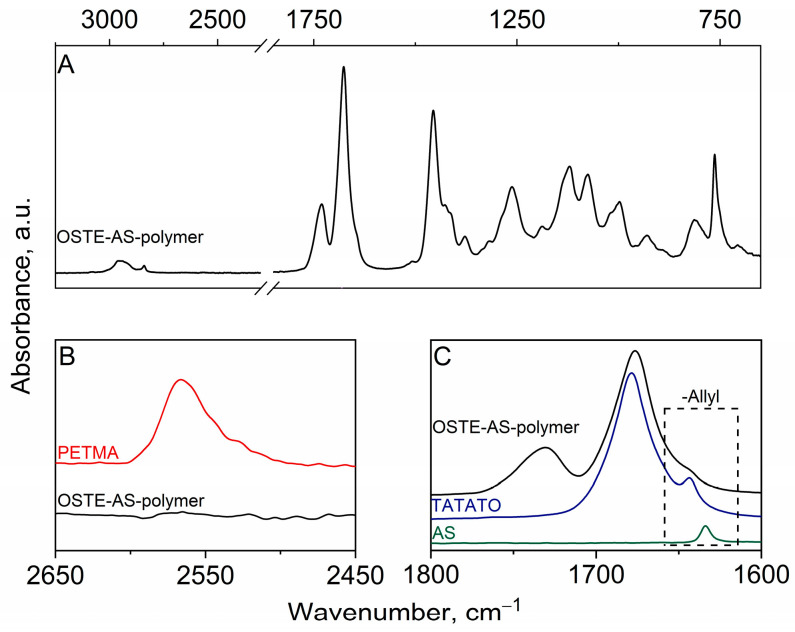
The FT-IR spectra of the OSTE-AS polymer: (**A**) full spectrum; (**B**) the region of thiol group bands; (**C**) the region of allyl group bands. Full spectra of TATATO, PETMA, AS, and prepolymer are presented in Supplementary Materials Figure S2.

The completeness of polymerization and the inclusion of allylsilanes in the polymer were determined using IR and NMR spectroscopy, respectively. During polymerization, characteristic bands disappeared in the IR spectrum at 2570 cm^−1^, corresponding to the thiol groups of PETMA, and at 1644 and 1634 cm^−1^, corresponding to the C=C fragments of TATATO and allylsilane, respectively (Figure 3). NMR studies were carried out using the extraction method. CDCl_3_ was chosen as the extraction solvent, since all the reagents that were used dissolve well in it. Only traces of the reagents were observed in the ^1^H NMR spectrum after extraction (see Supplementary Materials Figure S1). The disappearance of the SH band in the IR spectrum, as well as the absence of PETMA signals in the NMR spectrum, suggests that PETMA was fully included in the polymer framework. In the IR spectrum, the C=C band of AS also disappeared completely, and the C=C band of TATATO decreased significantly; furthermore, their signals were only present in the NMR spectrum in trace amounts. This suggests that AS and TATATO were also fully included in the polymer but that some of the allyl groups of TATATO remained free (roughly 15% due to overlapping signals in the IR spectrum).

### 3.2. Optimization of Polymer Composition

Variations in the reagent ratio have a strong effect on the mechanical properties of polymers. Therefore, in the next stage of this study, the polymer composition was optimized to achieve the maximum hardness, the maximum tensile strength, and a better bonding of the polymer to the silicon wafers. In order to simplify the procedure for the optimization of the polymer composition, first, the hardness, elasticity, and tensile strength were measured at various PETMA and TATATO ratios and at a fixed concentration of allylsilane. After selecting the optimal ratio of PETMA and TATATO, it was fixed, and the optimal silane concentration in the polymer was selected.

**Figure 4:**
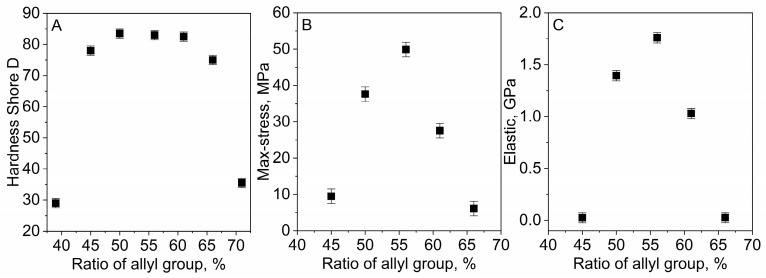
Dependence of (**A**) Shore hardness (type D), (**B**) max stress, and (**C**) elasticity on the ratio of allyl groups in the OSTE-MS prepolymers.

When measuring the hardness, the best results were obtained with a concentration of allyl groups (the number of allyl groups in the prepolymer mixture relative to the sum of allyl and thiol groups) in the OSTE-AS ranging from 45 to 65%. A decrease or increase in the concentration of the allyl groups in the prepolymer led to a significant decrease in the hardness of the resulting polymer (Figure 4A). The hardness of the OSTE-AS polymer is close to the hardness of the previously obtained OSTE-MS polymer [23]. A similar pattern was observed when studying the tensile strength; the highest maximum stress was observed for a close-to-equimolar ratio of the allyl and thiol groups in the prepolymer, and an imbalance towards an increase in the concentration of allyl or thiol groups led to a decrease in the maximum stress (Figure 4B). A diagram of the Young’s modulus is shown in Figure 4C, demonstrating a characteristic dependence on the maximum in the region of the close-to-equal concentration of the allyl and thiol groups in the prepolymer. It is worth noting that the Young’s modulus of OSTE-AS polymers varies over a large range of values (almost one order of magnitude), which makes it possible to obtain the elasticity required for a particular product or to even combine different OSTE-AS polymers in one product. For example, it is possible to obtain hard walls to make channels and soft elements to create valves. It is also possible to obtain polymers saturated with free thiol or vinyl groups. Such OSTE polymers can be easily modified using various functional compounds [25,26,27,28,29]. The obtained Young’s modulus and tensile strength values are close to the maximum values obtained for the OSTE-MS polymer, and they are equal to 1.7 GPa and 58 MPa, respectively [23]. The slightly higher value of the Young’s modulus of the OSTE-AS polymer (1.76 GPa) than that of the OSTE-MS polymer may be due to the presence of a smaller number of “linear” fragments (see Introduction), leading to its lower elasticity. However, there are few such fragments, so the difference in the values of the Young’s modulus is small. The Young’s modulus value of OSTE polymers containing epoxy modifiers is usually greater and ranges from 1.7 to 2.4 GPa [30].

In this work, it was decided to use a composition containing equal volumes of PETMA and TATATO monomers. This composition is easy to use (the error when mixing reagents is minimal) and is close to the maximum values of hardness and max stress due to the close-to-equimolar ratio of the allyl and thiol groups in the prepolymer. All further studies were carried out for polymers with an equal volume ratio of PETMA and TATATO.

**Figure 5:**
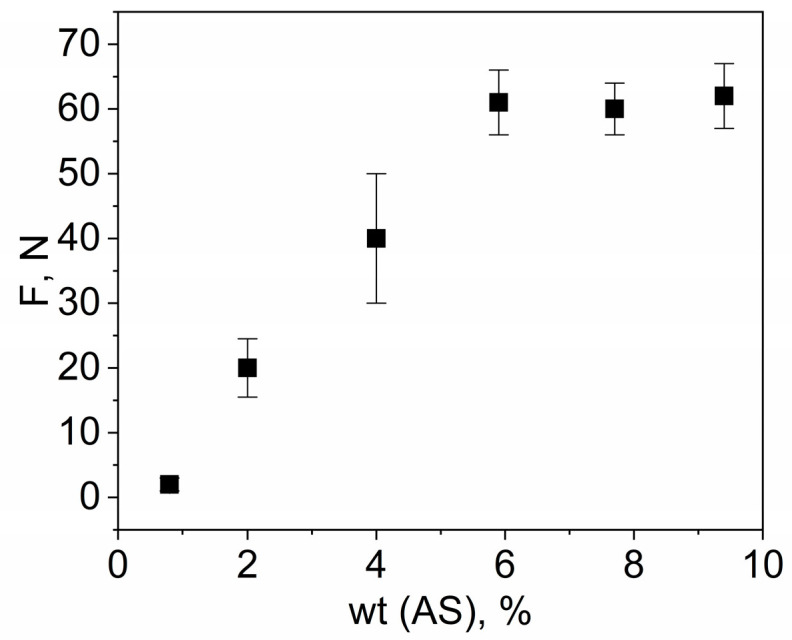
Dependence of the shift force on the concentration of allylsilane in the OSTE-AS polymers.

In our previous work, it was shown that an increase in the proportion of mercaptosilane in the polymer led to a decrease in the tensile strength but that, at the same time, the polymer bonded better with silicon wafers [23]. Similar properties were assumed for alkylsilane. Therefore, it was necessary to select the minimum required silane concentration for good bonding to the silicon wafers but with the preservation of acceptable mechanical properties of the polymer. When binding a polymer to a silicon wafer, it is important to compress them together. This is necessary to create good contact, in which case silane groups can react with surface hydroxide groups via the same mechanism as in the case of the formation of the thin films of silanes [31]. The binding strength was evaluated, as in previous work, by examining the shift force detachment of the polymer column from the silicon wafers. At low concentrations of allylsilane, a rapid increase in the shift force dependent on the silane concentration occurred; however, after about 5.9 wt%, the shift force almost stopped increasing (Figure 5). It is worth noting that the binding of the OSTE-AS polymer to the silicon wafers was greatly influenced by the pretreatment of the surface and the time between operations. Thus, the best data were obtained with a preliminary 10 min treatment of the silicon wafers in the UV/ozone to increase the number of hydroxide groups on the surface and by immediately adding a prepolymer to the wafer. An increase in the time between these operations led to a decrease in the shift force of 10–20 N.

The concentration of allylsilane also affected the wettability of oxide surfaces by the prepolymer and the viscosity of the prepolymer. The contact angle of the OSTE-AS prepolymer on a silicon wafer (with a natural oxide) decreased with increasing silane concentrations. Thus, the contact angles for the OSTE-AS prepolymers with silane concentrations of 0.8, 5.9, and 9.8% were 34, 24, and 15°, respectively (see full data in Table S1). The good wettability of the silicon surfaces caused by the OSTE-AS prepolymer allows them to be deposited onto a silicon wafer via centrifugation without the use of an additional adhesive. When using 100 mm silicon wafers, the thickness values of the OSTE-AS polymer (6:6:1 vol) films obtained via spinning were 12.9, 4.9, and 3.4 µm at 1000, 3000, and 5000 rpm, respectively. The compatibility of the polymer layer formation process with the technological equipment within the framework of chip production significantly expands the area of potential application of the developed OSTE-AS polymer in microelectronics. The viscosity of the OSTE-AS prepolymer also decreased with increasing silane concentrations. Therefore, at 20 °C, the viscosity of the prepolymer without the addition of silane was 0.61 Pa·s; at 2% silane, it was 0.45 Pa·s; and at 5.9%, it was 0.27 Pa·s (the data for various concentrations and temperatures are presented in Supplementary Materials Table S2). Thus, with an increase in the silane concentration in the OSTE-AS prepolymer, thinner films can be obtained via centrifugation.

Thus, in order to achieve a good binding of the polymer to silicon wafers and the maximum values of hardness and the maximum stress of the polymer, the composition of the prepolymer mixture was chosen, consisting of an equal volume ratio of PETMA and TATATO and 5.9% of the mass of allylsilane (or 6:6:1 volume ratio). All further experiments were carried out with the given polymer composition.

### 3.3. Physicochemical Properties of OSTE-AS Polymer

**Figure 6:**
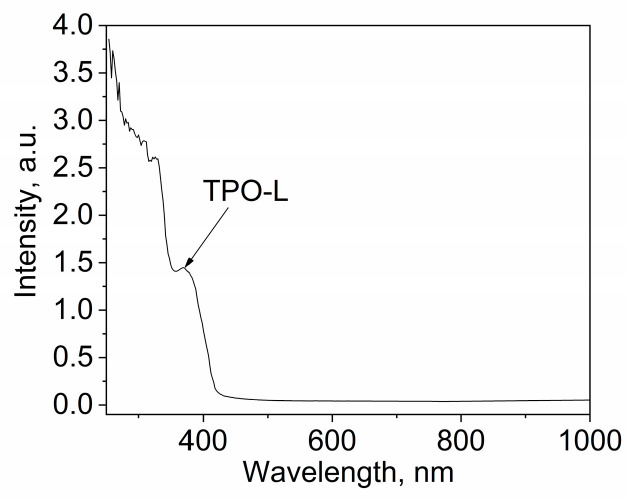
UV light absorption spectra of OSTE-MS polymer.

To use the developed polymer in various sensors and chip packaging applications, it is important to determine its light absorption, water wettability, dielectric constant, operating temperature ranges, and chemical resistance. OSTE-AS polymers, like OSTE-MS polymers, are transparent in the visible part of the spectrum and are limited in the near-ultraviolet region by the absorption peak of the TPO-L photoinitiator (Figure 6). The contact angle of the polymer is 70°, which is close to that of other OSTE polymers [32].

The dielectric constant of the polymer was calculated by measuring the capacitance–voltage characteristics of the capacitors that contained the OSTE-AS polymer as the dielectric layer. One of the capacitor plates was a silicon wafer, on which the OSTE-AS polymer was applied via centrifugation. Gallium was chosen as the second capacitor plate, and it was melted and deposited via dropping. This method of capacitor formation made it possible to obtain a good fit between the plates and the dielectric. The measurements were carried out at different thicknesses of the dielectric and plate areas. The average value of ε was 4.2, which is comparable to that of many polymeric materials, such as HDPE, Kapton, nylon, and silicone (RTV) [33].

**Figure 7:**
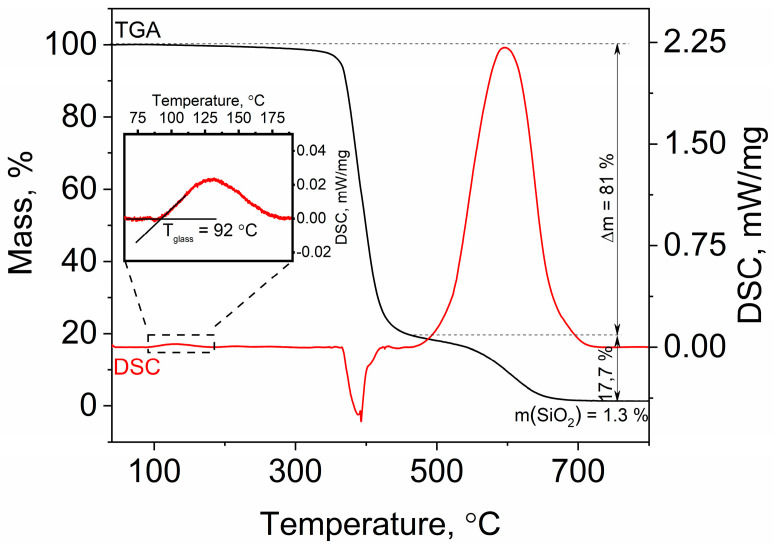
TGA and DSC curves of OSTE-AS polymer in air. Heating rate: 10 °C/min.

The measurements of the temperature dependences carried out using TGA and DSC showed that the OSTE-AS polymer had a glass transition temperature of 92 °C (Figure 7). The polymer was stable up to about 320 °C. With further heating, the endothermic decomposition of the polymer occurred, with a loss of approximately 81% of the mass at 474 °C. The rest almost completely burned out in air at 700 °C. The remaining 1.3% of the initial mass was SiO_2_, which formed from allylsilane. A similar behavior was observed for OSTE polymers without modifiers. In the case of OSTE polymers with epoxy modifiers, more char was formed during pyrolysis [30].

The chemical resistance of the OSTE-AS polymer to the main organic solvents used in chemical laboratories was evaluated. The assessment was carried out according to four main parameters: changes in mass, size, hardness, and visual control. The solvents were divided into four main groups according to the strength of their impact on the polymer (Table 1). The first group comprised the solvents that had almost no effect on the OSTE-AS polymer, even after 7 days of conditioning. Therefore, the chemical resistance of the polymer to this group of solvents was called “good”. This group comprised methanol, ethanol, propanol-2, hexane, white spirit, toluene, carbon tetrachloride, benzene, acetic acid, and ethyl acetate. When the OSTE-AS polymer interacted with the second group of solvents, the polymer slightly swelled after a week of treatment, and the mass and size increased. The chemical resistance of the polymer to this group of solvents was designated as “normal”. This group comprised butanone-2, THF, acetone, and DMSO. The chemical resistance of the polymer to the third group of solvents was designated “satisfactory”, and this group comprised DMF and acetonitrile, which led to the swelling of the OSTE-AS polymer after a day; after a few more days, they led to the degradation of polymer products. The chemical resistance of the polymer to the last group of solvents was designated as “unsatisfactory”, and this group comprised chloroform and methylene chloride, which led to the degradation of articles made of OSTE-AS polymers in less than a day. The complete data on chemical resistance are presented in Supplementary Materials Tables S3–S5.

It should be noted that over time, the polymer became more resistant to the solvents, even methylene chloride. The same behavior of OSTE polymers is described in the literature during prolonged heating to 100–200 °C [34]. This may be due to the additional formation of intrapolymer bonds, which initially did not form due to steric factors; however, after heating or a long waiting time, the functional groups appeared next to each other, which led to the formation of new bonds.

### 3.4. Creation of Microfluidic Systems

**Figure 8:**
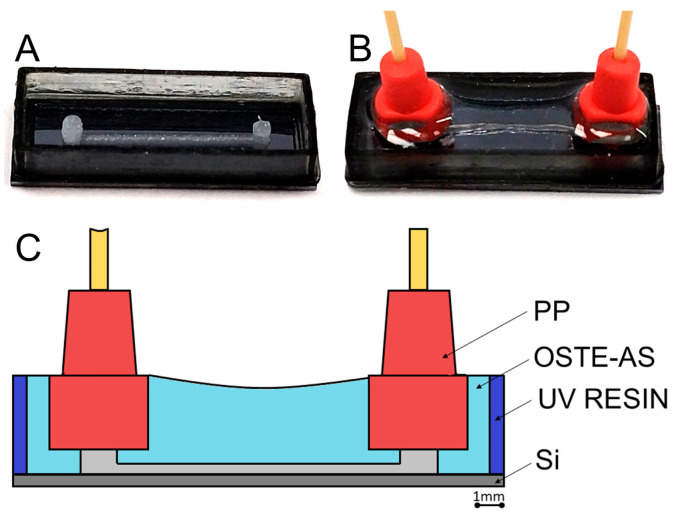
Formation of a microfluidic system on a silicon wafer: (**A**) preparation of a sacrificial layer; (**B**) finished microfluidic system; (**C**) cross section of the microfluidic system.

The practical application of the developed polymer was demonstrated by the creation of a microfluidic system on a silicon wafer. To create a microfluidic channel, we used the technology developed in our laboratory when creating a microfluidic system based on epoxy resins [35]. Using this technology, a sacrificial layer of Vaseline–wax ink was prepared in advance, after which the compound was added. After the curing of the compound, the sacrificial layer was removed. The outer frame can also be formed using Vaseline–wax ink; however, this process is time-consuming due to the slow printing speed of Vaseline–wax ink. To speed up the process of creating a microfluidic system, a fixed frame was used, which was previously printed using an SLA 3D printer (Figure 8A). Commercially available plastic connectors were added to the outlets to make it easier to attach tubing to the MFS. After the curing of the OSTE-AS polymer and heat treatment (with the compression of the polymer and silicon wafer), the Vaseline–wax ink was removed via washing with white spirit and hexane (Figure 8B).

The resulting microfluidic system showed good operational properties. It did not degrade over time, and liquid leaks (water and various buffer solutions were used) were not observed.

## 4. Conclusions

In this work, new off-stoichiometry thiol–ene polymers containing silane groups, which were included using allylsilane (modification by a thiol monomer), were obtained. An optimized polymer composition containing a 6:6:1 volume ratio of PETMA, TATATO, and AS, was selected due to its maximum hardness, tensile strength, and good bonding to silicon wafers. The Shore hardness (type D) of an optimized OSTE-AS polymer was 83, the tensile strength was 50 MPa, the Young’s modulus was 1.76 GPa, the contact angle of the water was 70°, and the dielectric constant was 4.2. The OSTE-AS polymer had a glass transition temperature of 92 °C and was stable up to 320 °C. It was optically transparent in the visible part of the spectrum. This polymer exhibited chemical resistance to the main organic solvents used in laboratories, except for chloroform and especially methylene chloride. The physicochemical properties of the OSTE-AS polymer are close to the properties of the OSTE-MS polymer studied in our previous work, which was developed via the reaction of mercaptosilane with an allyl monomer. The ability to choose the monomer interacting with the silane group allows for a more flexible selection of silane-containing OSTE polymers for the production of thiol- or allyl-excessive pieces. On the basis of the OSTE-AS polymer, a microfluidic system was obtained. The developed polymer can be widely used in microelectronic and biomedical industries as a material for packaging and as systems for delivery of analytes to sensors.

## Figures and Tables

**Table 1:** The chemical resistance of OSTE-AS to organic solvents at room temperature.

Solvent	Chemical Resistance ^1^
Methanol	Good
Ethanol	Good
2-Propanol	Good
Hexane	Good
White spirit	Good
Toluene	Good
Tetrachloromethane	Good
Benzene	Good
Acetic acid	Good
Ethyl acetate	Good
2-Butanone	Normal
Tetrahydrofuran	Normal
Acetone	Normal
Dimethyl sulfoxide	Normal
Acetonitrile	Satisfactory
Dimethylformamide	Satisfactory
Chloroform	Unsatisfactory
Dichloromethane	Unsatisfactory

^1^ See the text for explanation of “good”, “normal”, “satisfactory”, and “unsatisfactory” chemical resistance parameters.

## Data Availability

Not applicable.

## Supplementary Materials

The following supporting information can be downloaded at https://www.mdpi.com/article/10.3390/polym15061329/s1, Figure S1: NMR spectrum of the extract of OSTE-AS after aging for 1 h (CDCl_3_).; Figure S2: FT-IR spectra of OSTE-AS polymer, OSTE-MS prepolymer, and reagents (TATATO, PETMA, and AS); Table S1: The contact angle of the OSTE-AS prepolymer on a silicon wafer in various allylsilane concentrations; Table S2: The viscosity of the OSTE-AS prepolymer with various allylsilane concentrations and temperatures; Table S3: OSTE-AS polymer size change during conditioning in various solvents; Table S4: OSTE-AS polymer weight change during conditioning in various solvents; Table S5: OSTE-AS polymer hardness change during conditioning in various solvents.
