# Supplementary material for: Off-Stoichiometry Thiol–Ene Polymers: Inclusion of Anchor Groups Using Allylsilanes
Source: Polymers (Basel). 2023 Mar 7;15(6):1329. doi: 10.3390/polym15061329 (PMC10059650; doi:10.3390/polym15061329)
Supplement: Supplementary file 1 [file polymers-15-01329-s001.zip › polymers-2228409-supplementary.pdf]

Supplementary Material

Off-stoichiometry thiol–ene polymers: inclusion of anchor groups using allylsilanes

Kirill Puchnin, Dmitriy Ryazantsev, Egor Latipov, Vitaliy Grudtsov and Alexander Kuznetsov

puchninkv@yandex.ru

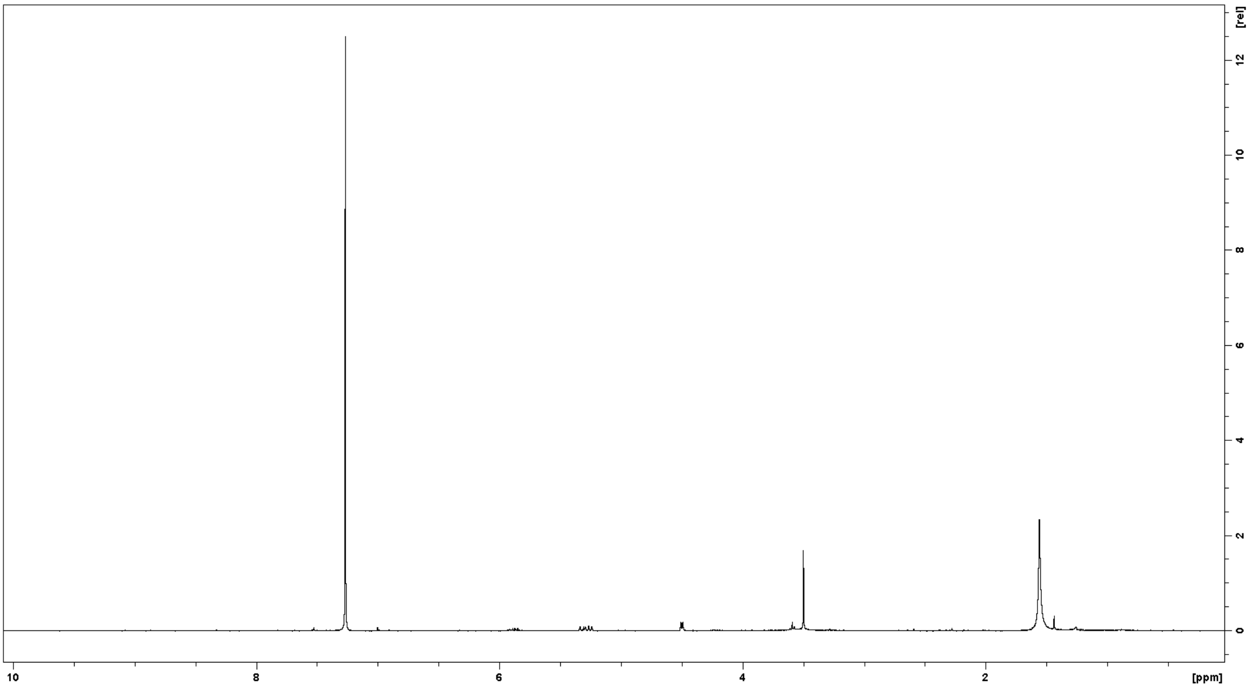

Figure S1. NMR spectrum of the extract of OSTE-AS after aging for 1 hour ($CDCl_3$).

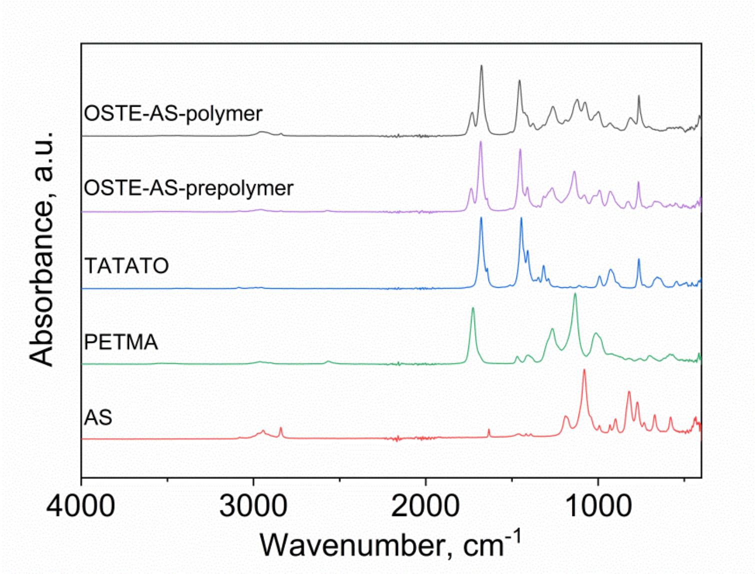

Figure S2. FT-IR spectra of OSTE-AS polymer, OSTE-MS prepolymer, and reagents (TATATO, PETMA, AS).

Table S1. The contact angle of the OSTE-AS prepolymer on a silicon wafer in various allylsilane concentrations.

wt (AS), %	Contact angle, °
0.8	34
2.0	32
4.0	28
5.9	24
7.7	23
9.4	15

Table S2. The viscosity of the OSTE-AS prepolymer with various allylsilane concentrations and temperatures.

wt (AS), %	Viscosity, Pa · s		
T, °C	15	20	25
0.0	0.86	0.61	0.44
2.0	0.62	0.45	0.33
4.0	0.46	0.35	0.27
5.9	0.35	0.27	0.22
7.7	0.28	0.22	0.19

Table S3. OSTE-AS polymer size change during conditioning in various solvents.

Solvent	Δl, %		
Time, h	1	24	168
Methanol	0.1	-0.2	0.4
Ethanol	0.7	0.5	0.5
2-Propanol	-0.6	-0.2	0.5
Hexane	-0.7	-0.1	-0.3
White spirit	0.0	0.2	0.4
Toluene	-0.4	-0.5	-0.2
Tetrachloromethane	0.0	0.1	0.1
Benzene	-0.1	-0.1	0.0
Acetic acid	-1.0	-0.7	-0.6
Ethyl acetate	0.4	0.0	0.4
2-Butanone	-0.5	0.6	3.1
Tetrahydrofuran	0.0	0.4	2.1
Acetone	-0.5	0.5	2.3
Dimethyl sulfoxide	0.2	0.7	2.1
Acetonitrile	-0.2	0.8	degradation
Dimethylformamide	0.2	1.8	degradation
Chloroform	0.6	degradation	degradation
Dichloromethane	1.3	degradation	degradation

Table S4. OSTE-AS polymer weight change during conditioning in various solvents.

Solvent	Δm, %		
Time, h	1	24	168
Methanol	0.0	0.0	0.2
Ethanol	-0.1	-0.1	0.0
2-Propanol	0.0	-0.1	-0.2
Hexane	0.0	-0.1	0.0
White spirit	0.1	0.0	0.0
Toluene	0.0	-0.1	0.1
Tetrachloromethane	0.0	-0.1	0.0
Benzene	0.0	0.0	0.3
Acetic acid	0.1	0.2	0.6
Ethyl acetate	0.1	0.3	1.0
2-Butanone	0.5	1.5	4.6
Tetrahydrofuran	0.4	1.7	5.3
Acetone	0.5	1.9	6.5
Dimethyl sulfoxide	0.5	2.4	7.3
Acetonitrile	1.0	3.7	degradation
Dimethylformamide	1.0	3.8	degradation
Chloroform	2.7	degradation	degradation
Dichloromethane	6.2	degradation	degradation

Table S5. OSTE-AS polymer hardness change during conditioning in various solvents.

Solvent	HD		
Time, h	1	24	168
Methanol	82.5	82.5	82.0
Ethanol	84.0	84.0	84.0
2-Propanol	83.0	83.5	84.0
Hexane	82.5	84.0	83.0
White spirit	83.0	82.0	84.0
Toluene	82.5	82.5	82.5
Tetrachloromethane	84.0	84.0	83.5
Benzene	83.5	84.0	83.0
Acetic acid	82.0	81.5	81.0
Ethyl acetate	84.0	84.0	83.5
2-Butanone	82.0	78.0	68.0
Tetrahydrofuran	81.0	79.5	73.0
Acetone	84.0	81.0	68.5
Dimethyl sulfoxide	83.5	81.0	73.0
Acetonitrile	81.0	75.0	degradation
Dimethylformamide	82.0	77.0	degradation
Chloroform	81.5	degradation	degradation
Dichloromethane	71.0	degradation	degradation
